# Brief Report on Primary Cutaneous Invasive Aspergillosis in a Patient With Prolonged Neutropenia Following a Traumatic Injury

**DOI:** 10.1093/ofid/ofaf140

**Published:** 2025-03-07

**Authors:** Armelle Pérez Cortés Villalobos, Ghadeer Alahmadi, Coleman Rotstein

**Affiliations:** Transplant Infectious Diseases, Multi-organ Transplant Program, University Health Network, Toronto, Ontario, Canada; Transplant Infectious Diseases, Multi-organ Transplant Program, University Health Network, Toronto, Ontario, Canada; Transplant Infectious Diseases, Multi-organ Transplant Program, University Health Network, Toronto, Ontario, Canada

**Keywords:** Aspergillosis, cutaneous aspergillosis, leukemia, neutropenia

## Abstract

Primary cutaneous aspergillosis is a rare fungal infection that usually arises from direct inoculation of the skin. We present a case of a patient with acute leukemia who sustained a traumatic injury to his finger, which progressed to a severe necrotizing soft tissue fungal infection following the onset neutropenia. This case underscores the importance of early recognition and prompt initiation of antifungal treatment in patients who are immunocompromised, while highlighting the potential for rapid progression and severe outcomes.

The incidence of primary cutaneous aspergillosis (PCA) in patients who are immunocompromised remains poorly defined but appears to be on the rise, likely due to enhanced reporting and the growing population of individuals with immunocompromised status. Here, we describe a unique case of PCA caused by *Aspergillus flavus* in a patient with acute myeloid leukemia who was experiencing prolonged neutropenia following induction chemotherapy. Notably, the patient had sustained trauma to the right fifth finger weeks before chemotherapy initiation, while the lesion remained clinically inactive without signs of cellulitis before the onset of neutropenia. Once chemotherapy-induced neutropenia developed, the lesion rapidly progressed to a severe necrotizing soft tissue infection, which ultimately required amputation despite aggressive treatment with liposomal amphotericin B. Subsequent improvement was achieved with voriconazole therapy.

This case highlights *A flavus* as a pathogen in PCA and underscores how a previously asymptomatic traumatic lesion rapidly progresses to a severe necrotizing infection in the setting of neutropenia. It also reinforces the importance of thorough history taking, as seemingly inactive wounds may become a nidus for invasive fungal disease. Clinicians should remain vigilant for PCA in patients who are neutropenic and immunocompromised with prior traumatic injuries.

## CASE

A 60-year-old male presented with febrile neutropenia and a necrotic fifth finger on his right hand. The patient's medical history was notable for multiple myeloma and autologous stem cell transplant 4 years before. He was recently diagnosed with chemotherapy-induced acute myeloid leukemia and was admitted for induction chemotherapy with fludarabine, high-dose cytarabine, idarubicin, and granulocyte-colony stimulating factor. He was persistently neutropenic and subsequently developed a febrile neutropenic episode caused by *Escherichia coli* bacteremia and *Candida guilliermondii* fungemia. For these bloodstream infections, he was treated with intravenous (IV) meropenem and liposomal amphotericin B. Of interest, despite his history of multiple myeloma and autologous stem cell transplantation, he had no prior invasive fungal infections (IFIs) requiring treatment. In addition, at the time of his induction chemotherapy for acute myeloid leukemia, he had not received any antifungal prophylaxis.

**Figure 1.**
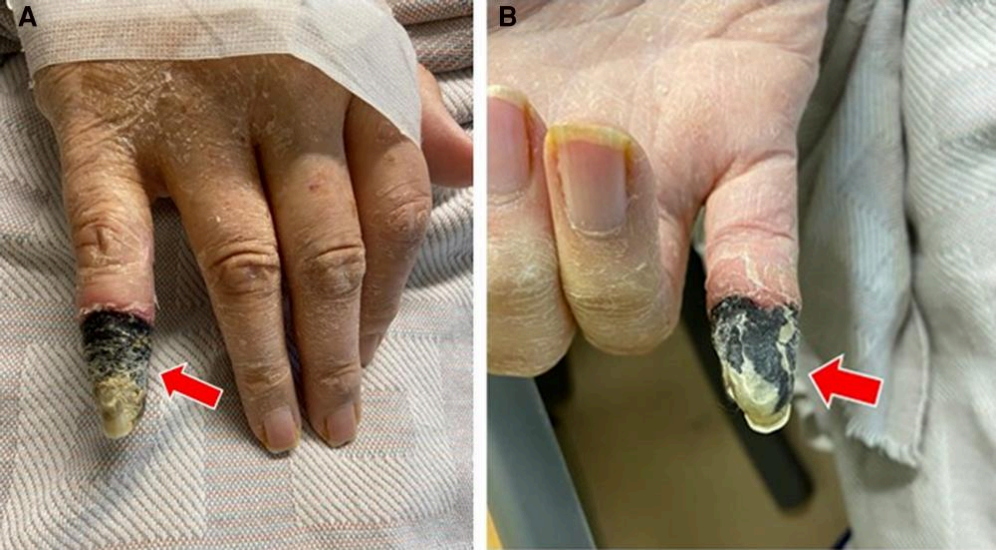
Primary cutaneous aspergillosis from right fifth finger shows complete necrosis of the finger: *A*, dorsal view; *B*, palmar view.

The physical examination revealed erythema, swelling, and severe pain in his right fifth finger. Because of this presumed cellulitis, IV daptomycin was added. The patient remained persistently febrile in the context of prolonged neutropenia, and after a week, the erythema and inflammation in his right fifth finger rapidly progressed to necrosis (Figure 1).

**Figure 2.**
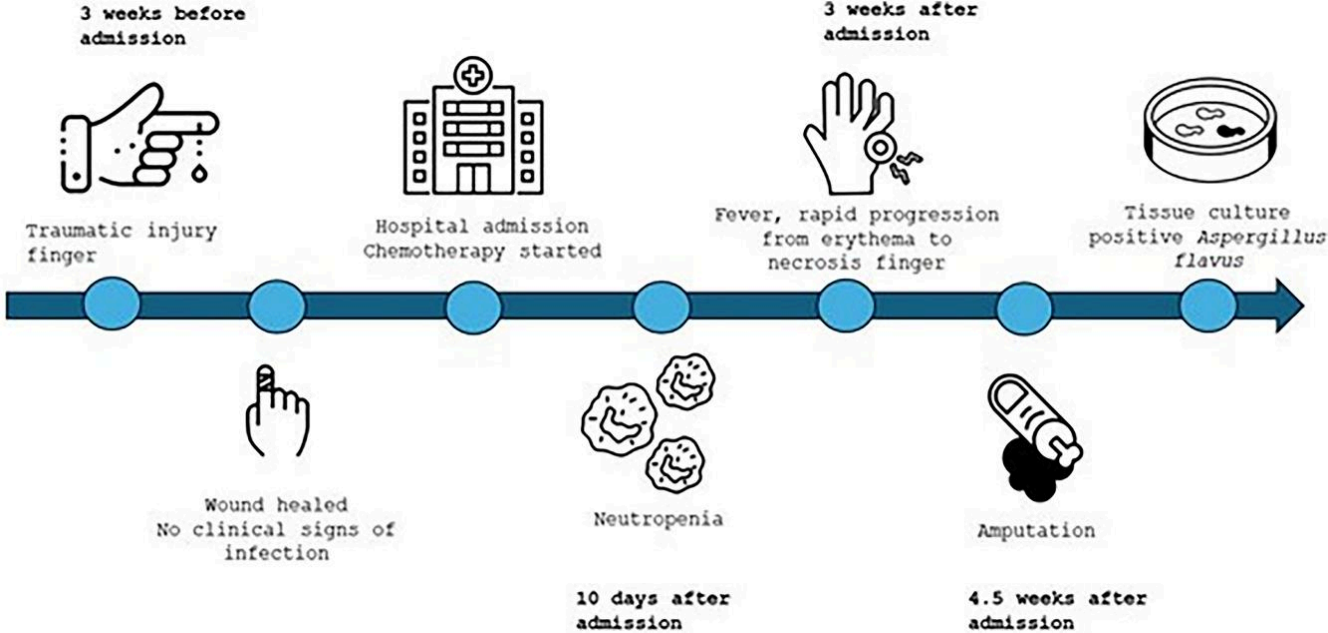
Visual timeline summarizing key events.

Upon further questioning, his history revealed that the patient had sustained trauma to his finger a few weeks prior to starting chemotherapy. He had accidentally traumatized his finger after being hit by a door, and he developed a small painless open wound in the finger that interestingly progressed to a severe necrotizing soft tissue infection during his prolonged neutropenic period. A computed tomography scan of the finger confirmed osteomyelitis of the distal and intermediate phalanges of the fifth finger, and given the severe progression of the infection, he underwent amputation of his right fifth fingertip at the proximal interphalangeal joint. A tissue specimen was sent to the clinical microbiology laboratory, where the bone culture confirmed *A flavus* infection. (Figure 2 illustrates the critical time points in disease evolution.) Unfortunately, no specimen was submitted for histopathologic examination. On the basis of these findings, amphotericin B was discontinued, and his antifungal therapy was transitioned to voriconazole as the agent of choice for *Aspergillus* infections. Since he was not undergoing antifungal prophylaxis, this PCA was not considered a breakthrough infection.

Subsequent computed tomography of the chest and abdomen showed no evidence of disseminated *Aspergillus* infection. After amputation of the finger, he was treated with voriconazole for 11 weeks before being transitioned to undertaking his allogeneic hematopoietic stem cell transplantation, and he remained afebrile with no signs of progression to disseminated aspergillosis. After the initiation of voriconazole therapy, drug monitoring was conducted for the first 14 days to ensure therapeutic levels, though not consistently thereafter. Unfortunately, he died after a failed bone marrow transplant.

## DISCUSSION

We present a case of PCA due to *A flavus* in a patient with a significant history of finger trauma sustained weeks before initiating chemotherapy, which progressed to an advanced localized fungal infection amid prolonged neutropenia. This case emphasizes essential insights into the importance of conducting a thorough medical and trauma history for patients scheduled for induction chemotherapy, particularly when severe neutropenia is expected. It illustrates the need to assess for any prior injuries and to maintain a high index of suspicion for IFIs in cases of necrotizing skin lesions that do not respond to antibiotics in the context of neutropenia.

Cutaneous aspergillosis is a rare but serious form of a locally invasive fungal disease that can present as a primary or secondary infection [1]. PCA typically arises at skin sites where direct inoculation has occurred—for example, near IV catheter insertion sites, areas with occlusive dressings, surgical wounds, burn sites, or areas of traumatic injury, as seen in our patient [2]. Secondary cutaneous lesions arise either from the direct spread of infection from underlying structures to the skin or through hematogenous dissemination, leading to skin involvement and often multiple lesions.

This patient had a clear history of a traumatic injury, and it is possible that there was direct fungal inoculation at the site of injury and subsequent angioinvasion, resulting in tissue necrosis [3]. The incidence of PCA in patients who are immunocompromised is a poorly described entity among bone marrow transplant recipients and patients with hematologic malignancies, mainly because literature reports have focused on severe disseminated *Aspergillus* infections. Nevertheless, it is clear that the common risk factor in this population of patients is recent neutropenia [4]. Some of the risk factors that have been identified are IV catheters, arm boards or tapes, and surgical wounds [5], all of which represent a disruption of the cutaneous and immunologic barrier in the context of neutropenia. In one report, a patient with multiple myeloma developed PCA after having some minor trauma with a plaster cast used to stabilized a pathologic fracture [4]. Additionally, there is a paucity of local phagocytic defenses permitting *Aspergillus conidia* to germinate, multiply, and invade the tissues.

The initial lesions of PCA may appear as macules, papules, nodules, or plaques [4]. Infections from occlusive tape used to secure a catheter may present with a hemorrhagic bulla [6], while infections originating from an IV catheter puncture typically manifest as skin erythema and induration, often progressing to necrosis [7]. Even though there can be inflammation, ulceration and the presence of pustules and purulent discharge have been described among neonates [8].

Another unique feature of this case report was the PCA involvement of the hand by *A flavus.* Epstein et al previously commented on the rare and unique association of *A flavus* with PCA of the hand, particularly in patients with hematologic malignancy [9]. However, one must be cognizant that the differential diagnosis of soft tissue infections in patients who are neutropenic is broad. Initial erythema often suggests a bacterial etiology, typically gram-positive organisms, but also gram-negative ones such as *Pseudomonas aeruginosa* that may cause necrotizing skin infections. Lack of improvement in response to potent broad-spectrum antibacterial agents, with progression to extensive necrosis, was a clue of another etiology in our patient. This pattern—localized devitalized tissue without hemodynamic instability—raised suspicion for an IFI.

Among fungal pathogens, *Fusarium* infections typically present as painful red or violaceous nodules with central ulceration and black eschar, often scattered across the trunk and extremities as the organism is typically blood borne. *Mucorales* infections, however, usually begin as painful erythematous lesions that rapidly evolve into black necrotic eschars with surrounding induration, tissue swelling, and pus formation, indicating extensive tissue destruction. Given the overlapping clinical features of IFI, microbiological confirmation is essential for identifying the causative pathogen and guiding appropriate treatment.

Even though this patient's cutaneous aspergillosis was localized, PCA can progress to disseminated infection in vulnerable hosts. For example, a reported case described a bone marrow transplant recipient whose PCA disseminated, ultimately resulting in a fatal outcome [10].

The diagnosis of PCA requires a skin lesion biopsy for culture and histopathology. It is recommended to take the biopsy from the center of the lesion, ensuring that it is deep enough to include subcutaneous tissue [4]. For histologic examination, routine fungal stains should include the Gomori methenamine silver stain, which highlights hyphae, since the hyphal cell wall stains black and has acute-angle branching and frequent septations. While these findings are characteristic of *Aspergillus*, they are not specific enough to confirm PCA. Due to variations in specimen sectioning, acute-angle *Aspergillus* branches may occasionally appear as right-angle branches, mimicking the right-angle branching and pauciseptated hyphae typical of zygomycete-like species. Therefore, a tissue fungal culture is also essential for accurate diagnosis. *A flavus* is a particularly important species in PCA according to previous reports [1, 5], and it accounts for 41.2% of cases of wounds infected by aspergillosis [11]. Other tests, such as serum galactomannan, have not been studied in PCA, and it is likely that there might not be additional value in monitoring with galactomannan [12]. After diagnosis of PCA, it is essential to assess the extent of the infection due to the angiotropic properties of *Aspergillus* spp. This evaluation should include investigations to rule out secondary seeding of infection.

Treatment usually consists of surgical debridement and antifungal therapy. The overall prognosis is significantly influenced by the patient's underlying immunologic condition.

In this case, the history of a traumatic injury to his finger was a crucial factor in raising suspicion of IFI. Notably, previously published cases of PCA share a common feature of direct skin trauma, including incidents related to tattooing [13], open fractures [14], or prior surgery [15].

It is relevant to recognize that invasive mold infections may occur in patients with neutropenia and other immunocompromised conditions following traumatic injuries. A high index of suspicion of an IFI should be triggered regarding skin lesions that quickly progress to necrosis or show no response to broad-spectrum antibacterial therapy. Heightened awareness of IFIs following traumatic injuries in patients who are immunocompromised is needed for early diagnosis and treatment to reduce dissemination and infection-related mortality.

## Notes

***Patient consent statement.*** The research board of the University Health Network does not mandate approval of a single patient case report. Instead, only reports involving 3 or more patients must receive board approval.

***Potential conflicts of interest.*** All authors: No reported conflicts.

## References

[1] Saghrouni F, Gheith S, Yaacoub A, et al Primary cutaneous aspergillosis due to *Aspergillus flavus* in a neutropenic patient. J Mycol Med 2011; 21:285–8.

[2] Murakawa GJ, Harvell JD, Lubitz P, Schnoll S, Lee S, Berger T. Cutaneous aspergillosis and acquired immunodeficiency syndrome. Arch Dermatol 2000; 136:365–9. 10724198 10.1001/archderm.136.3.365

[3] Giacobbe DR, Vena NRA, Bassetti M. Mould infections of traumatic wounds: a brief narrative review. Infect Dis Ther 2020; 9:1–15. 32072492 10.1007/s40121-020-00284-8 PMC7054562

[4] van Burik JA, Colven F, Spach DH. Cutaneous aspergillosis. J Clin Microbiol 1998; 36:3115–21. 9774549 10.1128/jcm.36.11.3115-3121.1998 PMC105285

[5] D’Antonio D, Pagano L, Girmenia C, et al Cutaneous aspergillosis in patients with haematological malignancies. Eur J Clin Microbiol Infect Dis 2000; 19:362–5. 10898138 10.1007/s100960050495

[6] Grossman ME, Fithian EC, Behrens C, Bissinger J, Fracaro M, Neu HC. Primary cutaneous aspergillosis in six leukemic children. J Am Acad Dermatol 1985; 12(2 pt 1):313–8. 3855880 10.1016/s0190-9622(85)80042-6

[7] Allo MD, Miller J, Townsend T, Tan C. Primary cutaneous aspergillosis associated with Hickman intravenous catheters. N Engl J Med 1987; 317:1105–8. 3657878 10.1056/NEJM198710293171802

[8] Gupta M, Weinberger B, Whitley-Williams PN. Cutaneous aspergillosis in a neonate. Pediatr Infect Dis J 1996; 15:464–5. 8724074 10.1097/00006454-199605000-00018

[9] Epstein MD, Segalman KA, Mulholland JH, Orbegoso CM. Successful treatment of primary cutaneous *Aspergillus flavus* infection of the hand with oral itraconazole. Hand Surg 1996; 21:1106–8. 10.1016/S0363-5023(96)80327-3 8969443

[10] Bretagne S, Bart-Delabesse E, Weschler J, Kuentz M, Dhedin N, Cordonnier C. Fatal primary cutaneous aspergillosis in a bone marrow transplant recipient: nosocomial acquisition in a laminar-air flow room. J Hosp Infect 1997; 36:235–9. 9253705 10.1016/s0195-6701(97)90199-7

[11] Pasqualotto AC, Denning DW. Post-operative aspergillosis. Clin Microbiol Infect 2006; 12:1060–76. 17002605 10.1111/j.1469-0691.2006.01512.x

[12] Verweij PE, Mennink-Kersten MASH. Issues with galactomannan testing. Med Mycol 2006; 44(suppl 1):S179–83. 30408901 10.1080/13693780600904918

[13] Zhang R, Zhang Y, Xu W, Han X, Zhao J. Primary cutaneous aspergillosis due to *Aspergillus fumigatus* in an immunocompetent patient with diabetes mellitus after tattooing: a case report and review of literature. Infect Drug Resist 2023; 16:791–7. 36779045 10.2147/IDR.S398858 PMC9911907

[14] Avkan-Oğuz V, Çelik M, Satoglu IS, Ergon MC, Açan AE. Primary cutaneous aspergillosis in immunocompetent adults: three cases and a review of the literature. Cureus 2020; 12:e6600. 32064182 10.7759/cureus.6600 PMC7008770

[15] Sharma S, Yenigalla BM, Naidu SK, Pidakala P. Primary cutaneous aspergillosis due to *Aspergillus tamarii* in an immunocompetent host. BMJ Case Rep 2013; 2013:010128. doi:10.1136/bcr-2013-010128 PMC3761659 23970496

